# Development of a Novel Design and Subsequent Fabrication of an Automated Touchless Hand Sanitizer Dispenser to Reduce the Spread of Contagious Diseases

**DOI:** 10.3390/healthcare9040445

**Published:** 2021-04-10

**Authors:** Arnab Das, Adittya Barua, Md. Ajwad Mohimin, Jainal Abedin, Mayeen Uddin Khandaker, Kholoud S. Al-mugren

**Affiliations:** 1 Department of Mechanical Engineering, Chittagong University of Engineering and Technology, Chittagong 4349, Bangladesh; arnabdasanik@gmail.com (A.D.); adittyabarua@yahoo.com (A.B.); ajwadmohimin@gmail.com (M.A.M.); 2 Faculty of Public Health, Thammasat University, Bangkok 10200, Thailand; abedinj88@yahoo.com; 3 Centre for Applied Physics and Radiation Technologies, School of Engineering and Technology, Sunway University, Bandar Sunway 47500, Selangor, Malaysia; 4 Department of Physics, Princess Nourah Bint Abdulrahman University, Riyadh 11144, Saudi Arabia; ksalmogren@pnu.edu.sa

**Keywords:** novel design, fabrication, automated dispenser, LDR based controller, reduction of COVID-19 spread

## Abstract

Background: The use of a touchless automated hand sanitizer dispenser may play a key role to reduce contagious diseases. The key problem of the conventional ultrasonic and infra-red-based dispensers is their malfunctioning due to the interference of sunlight, vehicle sound, etc. when deployed in busy public places. To overcome such limitations, this study introduced a laser-based sensing device to dispense sanitizer in an automated touchless process. Method: The dispensing system is based on an Arduino circuit breadboard where an ATmega328p microcontroller was pre-installed. To sense the proximity, a light-dependent resistor (LDR) is used where the laser light is to be blocked after the placement of human hands, hence produced a sharp decrease in the LDR sensor value. Once the LDR sensor value exceeds the lower threshold, the pump is actuated by the microcontroller, and the sanitizer dispenses through the nozzle. Results and discussion: A novel design and subsequent fabrication of a low-cost, touchless, automated sanitizer dispenser to be used in public places, was demonstrated. The overall performance of the manufactured device was analyzed based on the cost and power consumption, and environmental factors by deploying it in busy public places as well as in indoor environment in major cities in Bangladesh, and found to be more efficient and cost-effective compared to other dispensers available in the market. A comprehensive discussion on this unique design compared to the conventional ultrasonic and infra-red based dispensers, is presented to show its suitability over the commercial ones. The guidelines of the World Health Organization are followed for the preparation of sanitizer liquid. A clear demonstration of the circuitry connections is presented herein, which facilitates the interested individual to manufacture a cost-effective dispenser device in a relatively short time and use it accordingly. **Conclusion:** This study reveals that the LDR-based automated hand sanitizer dispenser system is a novel concept, and it is cost-effective compared to the conventional ones. The presented device is expected to play a key role in contactless hand disinfection in public places, and reduce the spread of infectious diseases in society.

## 1. Introduction

At present, the whole world is going through a pandemic due to coronavirus disease (COVID-19), which was first spotted in December 2019 in Wuhan, China. Since this virus is highly contagious, the World Health Organization (WHO) [1] has provided some guidelines to reduce its community transmission in various ways. One of the mandatory recommended actions is to perform hand washes/rub with soap/hand sanitizer in a frequent manner [1].

In principle, hand hygiene is now recognized as one of the most crucial issues for infection prevention and control. In the wake of the increasing severity of disease and treatment complexity, and a global pandemic superimposed by multidrug resistant (MDR) pathogen infections, the healthcare professionals (HCPs) are now returning to the basics of infection prevention by simple measures such as hand hygiene [2]. A relevant study conducted by White et al. [3] has shown a decrease of 14.8–39.9% in the upper respiratory disease symptoms among residential students (university) due to a general improvement of hand hygiene behavior.

Alcohol-based hand sanitizer (ABHS) is a useful material against the spread of infectious viruses in crowded areas such as clinics, workplaces, schools, etc. [2] It also helps to reduce the spread of disease-causing germs and bacteria. Early comprehensive research on the effectiveness of antiseptic hand rubs revealed that ABHS significantly reduces bacterial counts on hands [3]. Ehrenkranz et al. [4] reported that the ABHS is more effective in preventing the hand transfer of Gram-negative bacteria than the bland soap hand wash.

The hand sanitizer dispenser plays a significant role to allow individuals to wash/rub their hands using ABHS while on the go. A study by Fournier et al. [5] reported that the use of a strategically positioned hand sanitizer dispenser was successful in raising hand hygiene activity from 1.52% to over 60%. A few types of dispensers such as mechanical, automated with pushbuttons, touchless, etc., are available to dispense the liquid or gaseous sanitizing materials. In public places including hospitals, the use of mechanical dispensers is found widespread. Since physical contact is mandatory for using mechanical dispensers, they are vulnerable to pathogen infection. By performing a study on the hospital-based mechanical hand sanitizer dispenser, Erief et al. [6] concluded that the infected person may contaminate the dispenser which may trigger hospital-acquired infection. Automated pushbutton hand sanitizer dispensers are usually deployed in healthcare facilities, but these devices often have the possibility of being contaminated and become a center for pathogens [7]. Based on some other earlier studies [6,7], it is clear that mechanical and electrical dispensers (having a pushbutton) are vulnerable as these can be contaminated with pathogens that cause hand-associated infections (HAI). Consequently, nowadays, automated touchless sanitizers are taking place in healthcare facilities, especially in developed countries [8]. As this dispenser does not require any human contact to operate, it can be very effective to stop the spread of infectious diseases if used carefully.

A sanitizer dispenser can be made touchless automatic in different ways since various types of sensors can be used to sense the proximity [8]. Generally, ultrasound sensors [9,10,11,12,13,14,15] and infrared sensors [16,17,18,19,20] are used to make a low-cost sanitizer dispenser, but they show poor performances in public places where there is a lot of noise. Some dispensers are based on infrared radiation (IR) sensors, but they show malfunctions especially on sunny days where sunlight intensity varies because of clouds or reflection from the ground. However, such drawbacks can be easily overcome by using a light-dependent resistor (LDR) or photoresistive light sensor. In the present study, a laser light is used to block other reflections of light in the photoresistive light sensor, and this method of laser-based proximity tracking using an LDR sensor has proven to be more effective and more user friendly while considered to be used in busy public places. It is true that a large number of very low-income populations are living in the so-called developing countries like Bangladesh, Afghanistan, Cambodia, Guinea, Haiti, Laos, etc., and most of the time, some public places like bus stands, train stations, raw markets, hospitals, etc., remain crowded in those countries. The common population usually does not have the capability or is careless to maintain individual sanitization in a frequent manner. In such a situation, a touchless automated hand sanitizer dispenser is essential to stop the spreading of pathogens. Fortunately, due to the advancement of science and technology, it becomes possible to locally fabricate a low-cost automated hand sanitizer dispenser, and such a low-cost device may be very effective to be deployed in public places and individual use as well.

After a thorough analysis of both the online and offline market authors found that photo resistor sensor-based dispenser devices are not available in the market. In most of the dispenser devices, an infrared sensor is used for reducing the complexity and some devices are based on ultrasonic sensors. Based on the authors’ knowledge, this unique concept of making dispenser devices using an LDR (light dependent resistor) sensor with a laser light has received less attention from the scientific community, thus forms the main subject matters of this study.

The main objective of this study is to facilitate the process of assembling and making a low-cost hand sanitizer dispenser, which is fully touchless and automated using laser detection technology. In this paper, a novel design of an automated hand sanitizer dispenser is proposed, and subsequently, fabricated using the low-cost components that are commonly available in almost every developing country. A photoelectric resistor (LDR) is used to detect human hands inside the laser detection chamber, and this sensor is perfectly compatible with both daylight and night. A comprehensive discussion of the fabricated device with respect to the conventional ones is presented to show the pros and cons of this device. Thus, this study may help to stop the COVID-19 transmission in densely populated developing countries where industrial/commercial dispensers are costly and not readily available.

## 2. Materials and Methods

**Figure 1:**
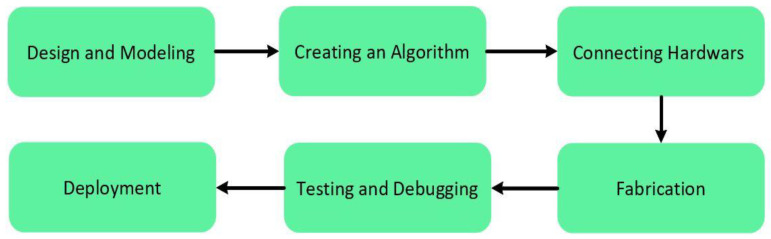
Flow diagram of the working process.

The main objective of this study is to develop an automated hand sanitizer dispenser that will be able to reduce the spread of viruses such as COVID-19 and save people from a pandemic. The dispenser device was fabricated under two key objectives: user-friendly and cost-effective. The materials to be used in the device fabrication were selected and actuated with these goals in mind. A brief information of the hardware components together with their key features is presented in Table 1. Furthermore, an overview of the fabrication process of the dispenser device is shown via a flow chart in Figure 1.

### 2.1. Design and Modeling

The performance of any device relies on different factors such as durability, the endurance of environmental change or it can be a totally psychological issue whether people are noticing the device or not. These factors mostly depend on the design or architecture of that device. The automated hand sanitizer dispenser device is designed in CAD software and the dimensions are shown in Table 2. The isometric view of the designed dispenser model is shown in Figure S1 with the dimensional parameters.

**Figure 2:**
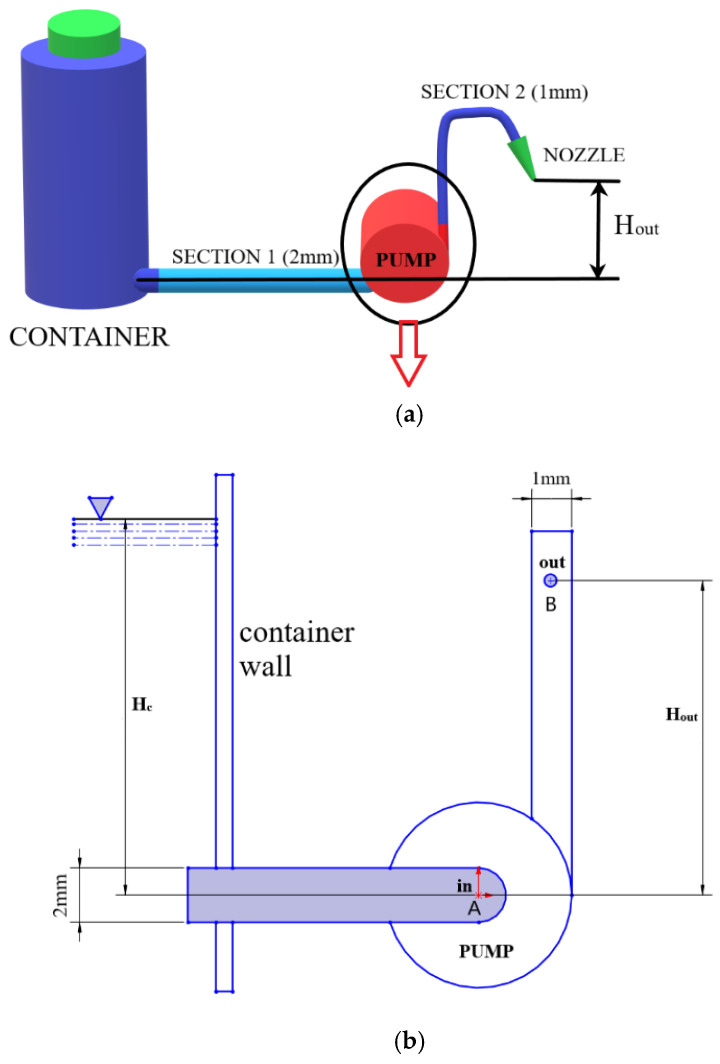
(**a**) Three-dimensional graphical representation of the piping system, (**b**) schematic diagram of the pump system

PVC flexible pipe was used in this work, which is sterile by a nontoxic, nonpyrogenic ethylene oxide (EO) gas. The pipe is divided into two sections; the diameter of the first section and the second section of the pipe is 3 and 2 mm, respectively. The first section is connected in between the motor pump and the sanitizer container, and the second section is connected to the outlet of the pump, which is directly connected to the spray nozzle/dispensing point. This nozzle is used to spray the sanitizer liquid. The two sections combined can supply 20 drops to 60 drops/mL, and it is disposable, also environmentally friendly. The rate of drops/mL is controlled by the pressure from the pump programmed into the microcontroller using Arduino IDE. The nozzle and piping system are commonly used as medical equipment for intravenous tubing for the rapid infusion of fluids. The piping system is shown in a block diagram (Figure 2a).

#### Theoretical Aspect

The dispenser device was designed based on some numerical assessment. The placement of the pipe in Section 1 was designed so that gravitational pull can be used to increase the fluid outlet pressure which helps to reduce the energy to be used by the motor pump to pump out the sanitizer. On the other hand, the placement of pipe in Section 2 was actuated by considering the loss of sanitizer from the nozzle due to the gravitational pressure and capillary pressure while the pump is off. This is ensured by optimizing the nozzle height based on the following calculations. The total pressure at the inlet of the pipe “Section 1” and outlet pressure at the end of the pipe “Section 2”, is calculated using Bernoulli’s Equations between the fluid (sanitizer) surface of the container and the fluid issuing from the nozzle [21].

(1) $$\frac{V^{2}}{2g}+\frac{P}{\gamma }+Z=constant$$

where *V* is the velocity of the dispensing media, *P* is the pressure at specific elevation, *ρ* is the fluid density, *g* is the acceleration due to gravity, $\frac{V^{2}}{2g}$ is the kinetic energy, $\frac{P}{\gamma }$ is the flow work, and *Z* is the potential energy. Here, losses due to the minor bending of the pipe are neglected. The schematic diagram showing the parameters is shown in Figure 2b.

The amount of energy required by the pump to process per unit weight of the fluid can be calculated by Equation (2).

(2) Z =  γQ(ΔH)

where *Q* is the volumetric flow rate and Δ*H* is the head imparted to liquid by the pump, and γ is the specific weight which can be calculated from

(3) γ = ρg

where ρ is the density of the sanitizer liquid and g is the acceleration due to gravity. The velocity at pump inlet point A and outlet point B is calculated using Equations (4) and (5).

(4) $$V{\mathrm{in}}_{\;}=\;\frac{Q}{Area}$$

(5) $$V{\mathrm{out}}_{\;}=\;{\left(\frac{r2}{r1}\right)}^{2}\times \;V_{\mathrm{in}}$$

Then applying Bernoulli’s equation to the free surface in the container and the inlet point A, taking the horizontal line through the inlet point A as the datum pressure at the pump inlet, Equation (5) gives:

(6) $$Hc_{\;}=\;\frac{Pin}{\gamma }\;+\;\frac{{\mathrm{Vin}}^{2}}{2g}{\;}_{\;}$$

Here, H_c_ is the distance of the centerline of the pump inlet pipe from the surface of the sanitizer inside the container. Energy per unit weight of liquid at outlet point B is greater than at A by the energy per unit weight supplied by the pump. Now applying Bernoulli’s equation between A and B, the pressure at the pump outlet is calculated from Equation (6),

(7) $$\frac{{\mathrm{Vin}}^{2}}{2g}+\frac{Pin}{\gamma }+\Delta H\;=\;\mathrm{Hout}+\frac{{\mathrm{Vout}}^{2}}{2g}+\frac{Pout}{\gamma }$$

Here, *P*_in_ and *P*_out_ are the inlet and outlet pressures and *H*_out_ is the height from the pump inlet pipe axis to the nozzle. Using the similar method, the pressure and velocity at the nozzle outlet is also determined, and based on these calculations the required height of the nozzle is calculated using the constant power output value of the motor pump.

### 2.2. Control System and Connection

Several control systems are available in the literature such as Arduino, Raspberry pie, Teensy 3.6, Particle photon, Adafruit Feather Huzzah, Beagleboard pocket Beagle, STM32F3 Discovery. However, in this study, the control system of the dispensing device was maintained by using Arduino software. The Arduino system consists of H-bridge, Arduino board, breadboard, pushbuttons, LED, and connectors. The main reasons behind the selection of the Arduino Uno system are its low cost compared to other systems, and the suitability to be used in cross-platforms. As a result, this system is compatible with every platform—Linux operating system, windows, Macintosh OS. While other control systems are only compatible with a particular system, either Linux or Windows, or Macintosh OS. Moreover, the supported signal bandwidth and RAM also show better features in Arduino than the other aforementioned control systems. Further favorable features are that it can be operated by a USB cable or a battery with a voltage range from 7 to 20 V(Volt). To protect the board from overload, and to control the 12 V motor pump properly, an L298 motor controller system was used. Figure S2 shows the overall connections between components with the Arduino board.

#### 2.2.1. L298 Controller Connection

**Figure 3:**
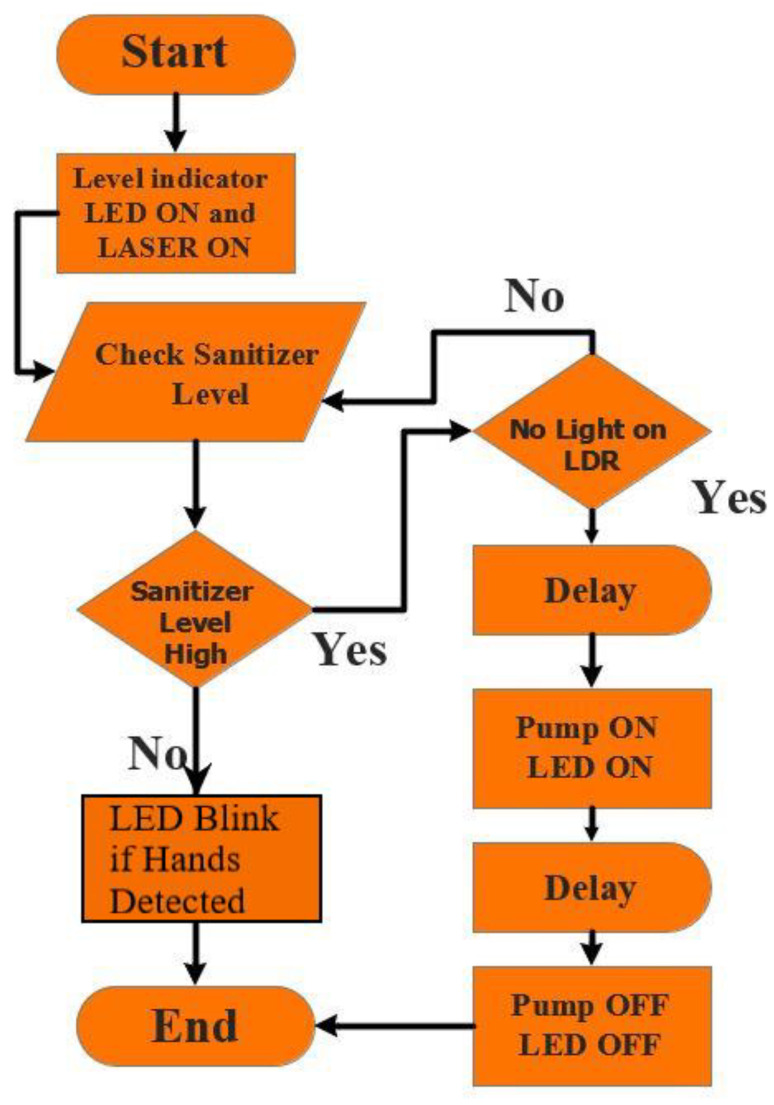
Original image of the proposed automated hand sanitizer dispenser; (**a**) placement of Arduino UNO, motor driver and pump, (**b**) setup of laser light, indicator LED, LDR sensor cabinet and nozzle, (**c**) sanitizer dispenser isometric view.

This system is powered by a 12 V DC input from the adapter through a 12 V port and GND port (see in Figure 3). It has an additional 5 V port besides these two ports. A 5 V capacitive laser is connected using this 5 V port and the GND port. The system has two enable (ENA) pins along with four input pins. These pins are connected to the analog and digital pins of the Arduino board. By these pins, the microcontroller is connected to the H-bridge of the L298 driver, and thus the motor pump can be controlled.

The motor controller has two output channels, and each channel was supplied 12 V DC. Any 12 V capacitive device can be connected using these output channels. In this study, the motor pump is connected by these channels (OUT3, OUT4). This type of connection arrangement provides an added advantage compared to conventional circuit connections. Generally, other devices operating within the range of 7–12 V can be powered directly from Arduino board (UNO). The ratings are listed below [22];

- The absolute maximum for any single IO pin is 40 mA (basically, it is the threshold at which Atmel can no longer guarantee that the chip will not be damaged);
- The total current from all the IO pins together is 200 mA max;
- The 5 V output pin is good for ~400 mA on USB, ~900 mA when using an external power adapter;
- The 900 mA is for an adapter that provides ~7 V. As the adapter voltage increases, the heat regulator has to deal with also increased values, so the maximum current will drop as the voltage increases. This is called thermal limiting.

The rating of the motor pump that is used in this study is 12 V–0.7 A; if the motor pump is directly connected to the Arduino board via GND pin and 5 V pin or other I/O pins three possible outcomes may occur:

- The pump will not run due to the low power supply;
- If the pump runs, the temperature of the voltage regulators will rise instantly and due to thermal limiting, the whole system will shut down temporarily;
- The motor pump will draw out the maximum threshold current and as a result, the chip can be damaged.

To overcome these problems L298 motor controller was used in this study which can supply DC 5 V–35 V; peak current 2 A. As a result, the motor pump can run continuously with a sufficient power supply.

#### 2.2.2. LDR Connection

The connection of the LDR photoresistor with the Arduino board is shown in the right panel of Figure 3. Here the LDR sensor is powered from 5 V and GND pins. A 10 KΩ resistor is connected with the GND line of LDR. Arduino measures voltages from analog pins A0 to A5. On the other hand, LDR is a variable resistor whose value changes based on the intensity of light, which cannot be measured by Arduino. Therefore, to convert the varying resistance to a voltage that can be measured by Arduino directly, the LDR is used with a resistor in a potential divider circuit.

The voltage at pin A0 is measured from:

(8) $$V_{0}\;=\;\frac{5*R1}{R1+R2}$$

Here, if the resistance value of the LDR (R2) varies, the voltage at pin A0 will also vary.

#### 2.2.3. Sanitizer Level Indicator

According to the algorithm of the present study, if the sanitizer level is lower than the threshold level, the whole process will stop. The main purpose of adding this system is to reduce power loss and also not to allow air bubbles inside the pipe through the container to pump inlet. Two probes are used here; one is connected to the 5 V pin of the Arduino circuit and the other probe is directly connected to the analog pin A1. The 5 V probe is placed at the bottom of the container and the other probe is placed just above the container outlet pipe. As a result, whenever the sanitizer level is above the limit, A1 gets a constant input signal. However, whenever below the limit, the A1-connected probe is disconnected and A1 gets “0” as a signal and the system recognizes this as the low sanitizer level.

### 2.3. Creating an Algorithm

**Figure 4:**
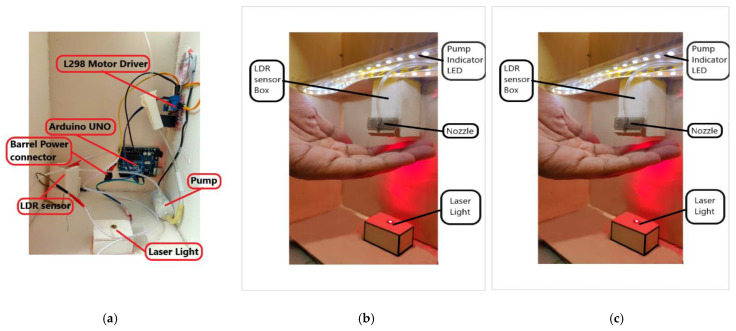
Flowchart of working pattern of the fabricated sanitizer dispenser.

An algorithm is what needed to be done before coding and fabrication. It is the step-by-step visualization of the total functionalities of the device. The working sequence of the device is depicted in a flowchart in Figure 4. A laser detection technology is used here to detect the object under the sensor and act accordingly. Whenever the LDR sensor detects a disturbance of the laser due to the hand of the user, the voltage of the photoresistor increases and the microcontroller can detect this phenomenon. Afterward, the microcontroller commands the motor driver to run the DC motor pump, which pumps out the desired amount of the sanitizing liquid from the container. Here, to ensure the desired functionality, the microcontroller is programmed according to the algorithm, considering the predictable accidental problems.

#### Programming, Coding, and Debugging

Arduino IDE was used in this study to program the atmega328P microcontroller. The Arduino IDE integrated development environment offers different libraries that can be used for programming. Library functions are simple and easy to use and do not require individual microcontroller registers to be addressed in the programming. The developed coding that is used in this study is shown in Figure S3.

Here, the limit 550 declared is the threshold limit for the LDR photoresistor which is set after various trials and errors in various lighting conditions. Motor pump speed control is introduced here. The pump start speed value is set to 10 initially which will gradually increase over time by adding 2 with the previous value. After that, the pump will stop instantly to stop the sanitizer flow instantly. Here, as the pump does not speed up instantly, the durability of the pump is increased.

After coding testing and debugging again and again this programme (shown in Figure S3) was made which was installed in the dispenser devices used in this study. The delay times were set after about 100 trial and error tests so that the run-time of the pump is perfect to pump out the required amount of sanitizer.

### 2.4. Fabrication Process

The fabrication and placements of the components are shown in Figure 3. To maintain the device’s stability, the pump was placed on the bottom surface of the container while the nozzle was placed at about ≤ half-height of the container’s height to take the advantage of liquid flow driven by gravity. Otherwise, the sanitizer may not get the needed pressure and the pump might not pump out the desired amount of liquids. The LDR sensor is kept inside a box or cabinet that is open to one side. Such an arrangement helps to make the nonfunctioning of the sensor due to the interference of natural sunlight or light from other sources such as vehicles, lamp posts, etc. This is a typical drawback of commercially available sensor-based dispensers, and some dispensers have a limitation to be placed only indoors because of the interference from unwanted light and sound signals (if an ultrasound sensor is used). An indicator LED strip was placed above the nozzle. This acts as an instruction for the user when to remove his/her hands from the laser detection chamber. Moreover, a cutout was made beside the transparent container and a LED strip was attached to the container. This action may help the user or maintenance authority to know the sanitizer level.

#### Preparation of Hand Sanitizer

At present, alcohol-based hand rubs are the only known means for rapidly and effectively inactivating a wide array of potentially harmful microorganisms on hands [23,24,25,26,27,28]. To help the countries and healthcare facilities, the World Health Organization (WHO) has recommended formulations for local preparation of alcohol-based hand rubs to be used for hand hygiene. Logistic, economic, safety, cultural, and religious factors have all been carefully considered by WHO before recommending such formulations for use worldwide. Hand sanitizer used in the automated touchless hand sanitizer dispenser developed in this study was prepared strictly following the WHO recommended formulation and procedure for local production [1], as shown in Table 3. The choice of components for the WHO-recommended hand rub formulations takes into accounts the cost constraints and microbicidal activity.

The required volume of ingredients (isopropyl alcohol, hydrogen peroxide and glycerol) was calculated using the following equation:

(9) $$\mathrm{Volume}\;\mathrm{of}\;\mathrm{starting}\;\mathrm{ingredient}\;\mathrm{required},\;(\mathrm{mL})\;=\;\frac{\left(Final\;\%\;of\;ingredient\right)\left(Final\;volume\;of\;preparation\right)}{Starting\;\%\;of\;ingredient}$$

Note: when the concentration of alcohol (e.g., ethanol or isopropyl alcohol) in the starting ingredient is not exact, the calculation should be adjusted accordingly to ensure a final concentration of at least 80% ethanol or 75% isopropyl alcohol.

Hand sanitizer for the dispenser developed in this study was prepared as of formulation 2, though both of the formulations can be followed depending on the availability of ingredients in the local market. By using a measuring cylinder, an amount of 7515 mL isopropyl alcohol (99.8%), 417 mL hydrogen peroxide (3%), and 145 mL glycerol (98%) were poured into a precleaned plastic bottle to prepare 10-L sanitizer. The bottle was then topped up with sterile distilled or cold boiled water to make a total volume of 10-L. The screw cap was placed on the bottle as soon as possible in order to prevent evaporation. The solution was then mixed thoroughly by shaking gently. An alcoholmeter was used to control the isopropyl alcohol concentration of the final solution. The concentration of hydrogen peroxide was measured by the titrimetric method (oxidation-reduction reaction by iodine in acidic conditions). The absence of microbial contamination (including spores) was checked by filtration, according to the European Pharmacopeia specifications [26]. National safety guidelines and local legislation were strictly followed in the purchase, transportation and storage of ingredients, and the final product.

## 3. Working Principle of the Dispenser

At first, the device should be plugged in using a 12 V AC-DC adapter. Then the process will automatically start to run without any human interaction. The functionality of this dispenser device is simple. Whenever the user puts his/her hand inside the laser detection chamber, the laser light is distracted (or intensity of light is reduced) due to the opaque media; human—hand. As a consequence, the voltage gain from the LDR sensor increases and the current flow through the LDR photoresistor decreases. Whenever the voltage gain exceeds the threshold value, the microcontroller acts according to the inserted program and sends a command to the motor driver to operate the pump and the indicator LEDs. 

In the working algorithm, a delay time was set before the microcontroller passes the trigger signal to run the pump. This initiative was taken to reduce the wastage of the liquid sanitizer in case of accidental disruption of the laser light signal. After the pump runs for a preprogrammed time, it turns off automatically and stops the flow of sanitizer. Then the process delays for a limited time (or the system becomes refreshed within a short time). Following the system revitalization, the loop starts again, and the machine becomes ready for rapid action. A delay time was also set between two consecutive full operations of the dispensing system, which also helps to reduce the wastage of sanitizer. 

A cutaway was included by which one can observe the sanitizer liquid level inside the container and refill whenever needed. For refill and maintenance purposes, a portion of the upper surface of the device was made foldable at about 120° angle. Two probes were inserted inside the container. These probes act as the sanitizer level indicator by which the microcontroller can check whether the liquid level is high or low. Whenever the sanitizer level goes down, the connection between two probes gets disconnected and it lets the microcontroller know that the sanitizer level is low. 

The running pump indicator LED strip is used to indicate that the pump is running and also it acts as an indication for users not to remove their hands from the chamber while the pump is still running. This initiative is also for reducing the wastage and also ensuring that the user gets the necessary amount of sanitizer needed to perfectly sanitize his/her hands. This indicator LED strip also indicates the low sanitizer level by blinking again and again, which is introduced in the code and also in the flowchart. As a result, the user may instantly know if there is sufficient sanitizer inside the container or not.

## 4. Results and Discussion

### Deployment and Performance Analysis of the Device

**Figure 5:**
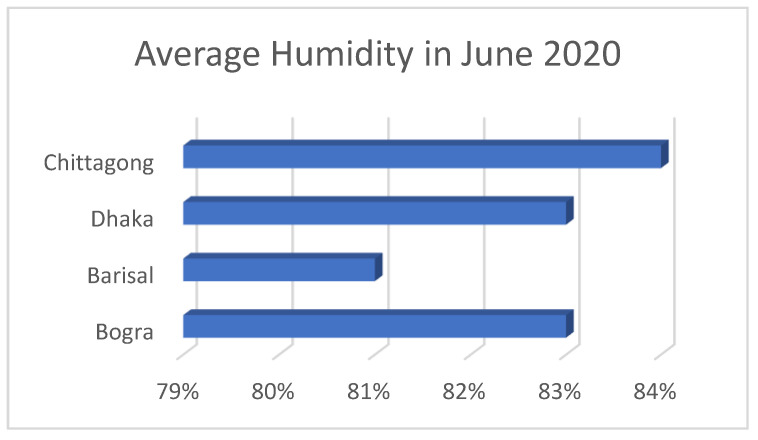
Average humidity comparison chart in June 2020.

A total of 20 units of fabricated devices, were deployed in four different metropolises in Bangladesh, and these regions have a clear variation in weather conditions. Figure 5 demonstrates the geographical locations of Bangladesh where the dispenser devices were deployed. Specifically, in each metropolis, several public locations such as hospitals, roadside bus stands, tea-stalls, varsity premises, etc., were chosen to deploy the devices. A list of the indoor and outdoor locations (where the devices were deployed) together with the GPS coordinates is shown in Table 4. These devices were deployed in the first week of June 2020, and their performances were analyzed from one to four months. Most of the deployed devices, that were fabricated according to the proposed design in this study, have shown an expected performance under the monitoring period.

Among 20 units of the dispenser devices, two units were found malfunctioned, another two units showed minor malfunctioning after a monitoring period of 1 month.

**Figure 6:**
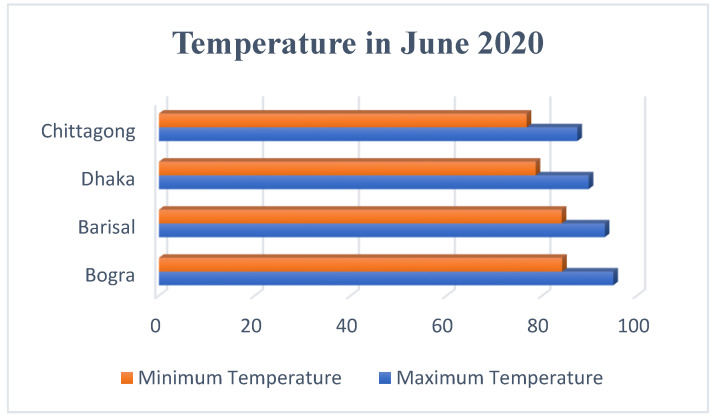
Maximum and minimum temperatures of the four districts in June 2020.

It was found that either the LDR sensor or the laser was damaged in the malfunctioned units. On the other hand, the disconnection of wires was observed in the minor malfunctioning units which could be the result of a transport problem or accidental shake or vibrations. For further confirmation of the reasons of malfunctioning, the malfunctioned units were analyzed by disassembling and reassembling the whole device. However, a number of external factors such as temperature, humidity, rainfall, UV index were identified as responsible for the damage of the sensor and laser light in the malfunctioned units. Analytics of these factors, particularly temperature and humidity are shown in Figure 5 and Figure 6, respectively.

Figure 6 shows the maximum and minimum of temperature of the studied region with the following order Chittagong < Dhaka < Barisal < Bogra, while the humidity of the studied regions show an order of Barisal < Bogra < Dhaka < Chittagong (see in Figure 5).

It can be assumed that the performance of the photoresistor and the laser was affected by the temperature and humidity. The two malfunctioned units (that were deployed outdoors) were found in Barisal and Bogra. Since these two cities show relatively higher temperature, it can be assumed that the LDR sensor or the laser was damaged because of higher daytime temperature or excessive sunlight. On the other hand, the two semi malfunctioned units were found in Chittagong city where they were deployed in the outdoor environment. Since a greater value of humidity was found in this city, such a high humidity might cause the partial malfunctioning of the devices via dampening the sensor surface which prevented the laser to penetrate. Excessive dust can also be a reason for the partial malfunctioning. These assumptions are based on the fact that all dispenser units that were deployed in the indoor environment showed smooth functioning even after four months of deployment. It is thus assumed that the devices that were deployed in the outdoor environment and showed a partial or full malfunction were because of either the laser and LDR damage or affected due to the excessive dust, temperature, or humidity.

A review of literature revealed that numerous dispenser devices of various designs and sensing techniques are available in the market. However, all of these devices are rather expensive for the general population, especially for the people of the third world countries where a great portion of the total population are living under the poverty line. However, in order to perform a cost-effective analysis, the commonly used automated dispenser devices are listed in Table 5.

The prices shown in Table 5 were taken from different online shopping platforms like amazon.com and Aliexpress which are accessible to most of the countries in the world. After a thorough analysis of the online market prices, the commercial dispensers show a price range from USD 29 to 180, where our fabricated dispenser shows a maximum cost of only around USD 25. As this device is made of components that are available in almost every country at a very low price, mostly available on online sites, it is helpful for normal people to make a dispenser of their own. The present device can be made more cost-effective if the Arduino and motor driver circuits are replaced by a custom-made circuit using a relay instead of a microcontroller.

Most of these dispensing devices are operated based on AA batteries. However, since these batteries discharge faster, they need to be changed quite frequently which adds extra expense. To minimize such expenses and also to ensure a constant good performance, a 12 V–1 A AC to DC adapter was used in our fabricated device. Compared to the dispenser from KENT and SaniQuick, the present device consumes much less power (only 12 W/cycle) without sacrificing an equal graded performance. Table 5 shows that most of the automated sanitizing devices do not use a specific pump rather they are operated by a DC volt battery. However, as these devices rely on gravity, the mechanical pumping system becomes malfunctioning after a certain time of use because of “Buoyant force” and “Capillary force”. Moreover, to take the advantage of gravity, these devices need to be set only at a position perpendicular to the ground.

Almost all of these devices used a custom-made valve to control the flow of the sanitizer; the working principle of this valve is mainly based on the gravitational pull. As a result, sometimes these devices face some leakage issues which can be avoided in the proposed designed dispenser as it is partially dependent on gravity but totally dependent on the pump.

**Figure 7:**
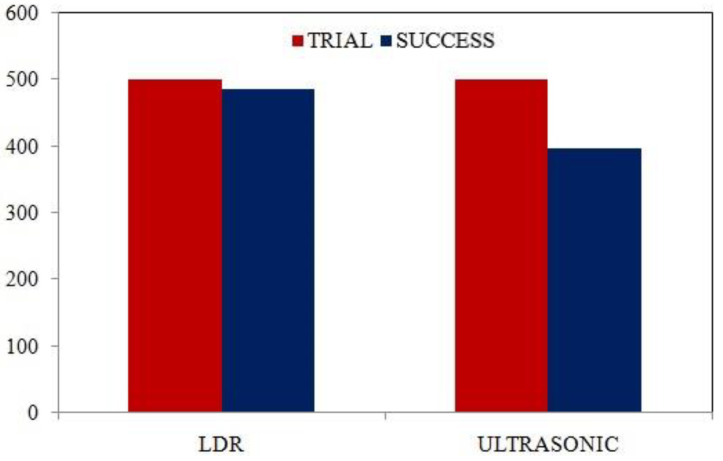
Comparison of trial-based success rate: (1) LDR sensor, (2) ultrasonic sensor.

The commercial devices (shown in Table 5) are using two types of sensors—infrared (IR) and ultrasound sensors. In general, the IR sensors show better accuracy than ultrasonic sensors. However, the IR sensors show some drawbacks like “Reading effects” by the infrared radiation of the sun light [29]. As a result, the IR sensor-based sanitizer dispensers cannot be deployed in an outdoor environment or in places having bright sunlight. On the other hand, to understand the effectiveness of our dispenser device compared to the ultrasound sensors, a real-time experiment was performed using the same experimental setup in this study. About 500 trials were recorded and after analyzing the results of the trial the outcome is shown in Figure 7.

Both devices with the LDR sensor and ultrasound sensor were placed in a crowded bus station (GEC Circle Bus Stop; 22°21′32.7″ N, 91°49′16.5″ E). From the column chart shown in Figure 7, it is crystal clear that for outdoor use, the ultrasonic sensor has shown underperformance compared to the current device of the laser detection system with an LDR sensor. One of the main drawbacks of the ultrasound sensor is that it provides garbage readings from different sound sources named the “Reading effect” [29]. As a result, the device sometimes runs automatically even if there is no user, consequently wasting the sanitizing material and making a mess underneath the device. 

In the present device, the use of laser detection by an LDR sensor helps to avoid the reading effects typically found in the ultrasonic sensor. The LDR sensor is placed inside a 7 cm tall cabinet so that the reflected sunlight or other light sources cannot disturb the performance of the device. Moreover, the default delay time between two working cycles is lower in the case of LDR than the ultrasonic. As a result, LDR sensors have higher response frequency and lower delay time. In the present device, the volume of the container is 1000 mL. Since an approximate amount of 1 mL sanitizer is dispensed through the nozzle in an operation cycle, therefore 1 L of the sanitizing material is sufficient to serve about 1000 people.

## 5. Conclusions

In this study, a novel design of an automated hand sanitizer dispenser was demonstrated. The components needed for the device fabrication were described in detail. The circuit diagram was discussed, which clarifies the connection between the components with the microcontroller circuit (Arduino UNO). The piping conditions were shown and described accordingly. The relevant diagrams and components of the original device were presented in sequential order for a better understanding of the device fabrication process/model. Arduino IDE was used to input the program into the microcontroller. The algorithm used in this device was described with a flowchart to depict the functionality of the dispenser. A comparison was made to assess the effectiveness of the current LDR sensor with the ultrasonic and IR sensor which are generally used for commercial purposes. Based on this study, our fabricated device shows the following advantages:

- Superior performances for indoor use;
- Consumes low power; while in standby mode it consumes only 0.05 mA, and during the operating cycle it consumes about 12 V and 0.5 A;
- Cheap readymade components like Arduino Uno, L298 motor driver, 5 V laser, 5 mm LDR are used. As a result, this device can be made at a low cost with a price range from about USD 20 to 25;
- LDR sensor is more efficient to be used for proximity sensing than ultrasonic sensor in the case of the outdoor use of this dispenser.

The automatic touchless hand sanitizer device demonstrated in this study is expected to play a key role in contactless hand disinfection in public places and reduce the spread of infectious diseases in society.

## Figures and Tables

**Table 1:** List of all components that were used to fabricate the automated hand sanitizer dispenser.

Major Components	Manufacturer/Brand	Advantages	Price (USD)
R3 Board ATmega328P with USB Cable for Arduino-Compatible	Kuman	Inexpensive Cross-platform-runs on Windows, Macintosh OSX, and Linux operating systems while other microcontroller circuits only run on windows User-friendly programming environment	10.690
MCIGICM Photoresistor Photo Light Sensitive Resistor, Light Dependent Resistor 5 mm	MCIGICM	Light resistance (at 10 Lux): 5–10 Kohm which is useful in this study Perfect dark resistance: 0.5 Mohm Good response time: 20 ms (Rise), 30 ms (Down) Resistance illumination: 4	0.133
HiLetgo^®^ L298N Motor Drive Controller Board Module Dual H Bridge DC Stepper For Arduino	HiLetgo^®^	Over-temperature protection Maximum supply voltage 35 V Maximum output DC current 4 A Low saturation voltage Logical “0” input voltage up to 1.5 V	2.470
Uxcell Female DC Power Jack Socket Connector	Uxcell	Easy to connect Barrel connectors are not rare and used universally	1.018
HiLetgo 5V 650 nm 5 mW Red Dot Laser Head Red Laser Diode Laser Tube with Leads Head Outer Diameter 6mm	HiLetgo^®^	Optimal temperature tolerance: −36~65 °C operating temperature range Low working voltage: 5 V DC	0.599
EDGELEC 100 K ohm Resistor 1/4 w (0.25 Watt) ± 1% Tolerance Metal Film Fixed Resistor	EDGELEC	Long working life High temperature resistance Moisture proof	0.057
JOVNO 12V 1A Power Supply Adapter 100–240 V AC to DC 12volt 12 W 1amp 800 mA 500 mA Power Converter Transformer with 5.5 × 2.5 mm Tip	JOVNO	Universal adapter: Built in automatic over-voltage protection, Over-current protection Over-temperature protection Short circuit protection Fire retardant shell	3.990
Zlolia 12 V Waterproof LED Strip Light 5 M 300LEDs	Zlolia	Waterproof Long life span: 50,000 h	0.010
Mini DC Brushless Water Pump JT-600C-12 16 mm Internal Thread	Zjchao	Long life and low noise, easy to install The pump is made of resin glue seal which is of good insulation	6.280

**Table 2:** Design parameters of the dispensing device.

Physical Quantity or Measurand	Parameters	Units (cm)
Basement	Length (l) × width (w)	20 × 20
Sidewall	Length, l_1_, l_2_	27, 32
Laser detection chamber	Height, h_c_	13

l_1_ = Height of the lower part of the left and right side wall of the device from the base, l_2_ = Height of the upper part of the left and right wall of the device from the basement, h_c_ = Height of the chamber where users may put their hand palms.

**Table 3:** World Health Organization recommended formulations for local production of alcohol-based hand sanitizer.

Formulation	Required Ingredients (Starting % of Ingredient)	Concentrations in Final Product, % (*v/v*), (Final % of Ingredient)	Required Volume of Ingredients for 10-L Preparation, mL
1	(i). Ethanol 96%	80	8333
	(ii). Hydrogen peroxide 3%	0.125	417
	(iii). Glycerol 98	1.45	145
	(iv). Sterile distilled water or boiled cold water	---	1105
2	(i). Isopropanol 99.8%	75	7515
	(ii). Hydrogen peroxide 3%	0.125	417
	(iii). Glycerol 98%	1.45	145
	(iv). Sterile distilled water or boiled cold water	---	1923

**Table 4:** Deployment of the fabricated hand sanitizer dispenser units in various major cities in Bangladesh.

Deployed Locations	Address	GPS Coordinates	Number of Machines	Placement Environment	Observed Working Condition
Shaheed Ziaur Rahman Medical College	Bogra City Bypass, Bogura 5800	24°49′40.6″ N 89°21′10.8″ E	1	Indoor	Working
Mohammad Ali Hospital	01 Sherpur Rd, Bogura 5800	24°50′08.2″ N 89°22′27.0″ E	1	Indoor	Working
Choumatha Markaj Jame Masjid	C & B Rd, Bhanga-Barisal Hwy, Barishal 8200	22°42′02.9″ N 90°21′11.4″ E	1	Outdoor	Malfunctioned
Chittagong Medical College Hospital	57 K.B. Fazlul Kader Rd, Chattogram 4203	22°21′33.7″ N 91°49′50.6″ E	8	Four Indoor Four Outdoor	Working One unit minor malfunction
Nagar Bhaban	Batali Hill, Tiger Pass, Chattogram	22°20′39.9″ N 91°48′51.3″ E	2	Indoor	Working
GEC Circle Bus Stop	GEC More, Chattogram	22°21′32.7″ N 91°49′16.5″ E	1	Outdoor	Minor malfunction
Askar Ali Jame Masjid	Gundip, Anowara, Chattogram	22°20’22.1″ N 91°84’33.1″ E	1	Outdoor	Working
Sadar Thana Bogura	Bogura	24°51’01.6″ N 89°22’22.1″ E	1	Indoor	Working
Satmatha Traffic Police Box	Park Rd, Bogra	24°50′50.8″ N 89°22′21.3″ E	1	Outdoor	Malfunctioned
Chatori Choumohoni Bazar	Gundip, Anowara, Chattogram	22°23′27.4″ N 91°87′18.1″ E	1	Indoor	Working
Bangladesh Awami League Central Office	23 Bangabandhu Ave, Dhaka	23°43′36.3″ N 90°24′40.4″ E	2	Indoor	Working

**Table 5:** Available dispenser devices name sensor power price.

Name	Price in (USD)	Sensor	Power Consumption/Cycle (W) P = VI
Bremmer Hand Sanitizer Dispenser Wall Mounted (1000 mL)	73.0583 Approx.	Infrared sensor	4 AA dry cell batteries. (1.5 V–0.5 Amp) 3 W
Zurio Automatic Hand Sanitizer Dispenser Wall Mount (450 mL)	59.99	Infrared sensor	12 V–0.5 A 6 W
JS LifeStyle Automatic Hand Sanitizer Dispenser (1000 mL)	29.99	Infrared sensor	3 AA battery (1.5 V–0.5 A) 2.25 W
Luxtonusa Automatic Hand Sanitizer Dispenser (500 mL)	179.95	Motion activated sensor	4 C-sized battery (1.5 V–3.8 A) 22.8 W
CasaTimo Automatic Touchless Hand Sanitizer Dispenser (450 mL)	35.99	Motion activated sensor	4 AA dry cell batteries. (1.5 V–0.5 Amp) 3 W
Safeline 360 Automatic Sanitizer/soap Dispenser (1000 mL)	59	Infrared sensor	4 AA dry cell batteries. (1.5 V–0.5 Amp) 3 W
Guukar Automatic Soap Dispenser Hand Sanitizer (500 mL)	41.99	Infrared sensor	4 AA dry cell batteries. (1.5 V–0.5 Amp) 3 W
Kent Auto Hand Sanitizer Dispenser (12,000 mL)	161.846 Approx.	Infrared sensor	Input: 230 V AC 24 V DC–1.5 A Total rated Consumption: 40 W
SaniQuick Touch Free Soap and Sanitizer Dispenser (3200 mL)	155.102 Approx.	Ultrasonic sensor	Input: 220 V AC

## Supplementary Materials

The following are available online at https://www.mdpi.com/article/10.3390/healthcare9040445/s1, Figure S1: 3D isometric view with parameters, Figure S2: Schematic of the dispenser circuit, Figure S3: The coding of the dispenser according to the algorithm.

## Nomenclature

V	Fluid velocity (m/s)
P	Fluid pressure (N/m^2^)
g	Gravitational acceleration (m/s^2^)
*γ*	Specific weight (N/m^3^)
Z	Potential energy per unit weight (J/kg)
Q	Volumetric flow rate (m^3^/s)
r	Radius (m)
Δ*H*	The head imparted to liquid by the pump (m)
H	Height (m)
V_0_	Voltage at A0 pin of Arduino (Volt)
R1	10 kΩ resistance
R2	Resistance value of LDR (kΩ)
