# Supplementary material for: Development of a Novel Design and Subsequent Fabrication of an Automated Touchless Hand Sanitizer Dispenser to Reduce the Spread of Contagious Diseases
Source: Healthcare (Basel). 2021 Apr 10;9(4):445. doi: 10.3390/healthcare9040445 (PMC8070052; doi:10.3390/healthcare9040445)
Supplement: Supplementary file 1 [file healthcare-09-00445-s001.pdf]

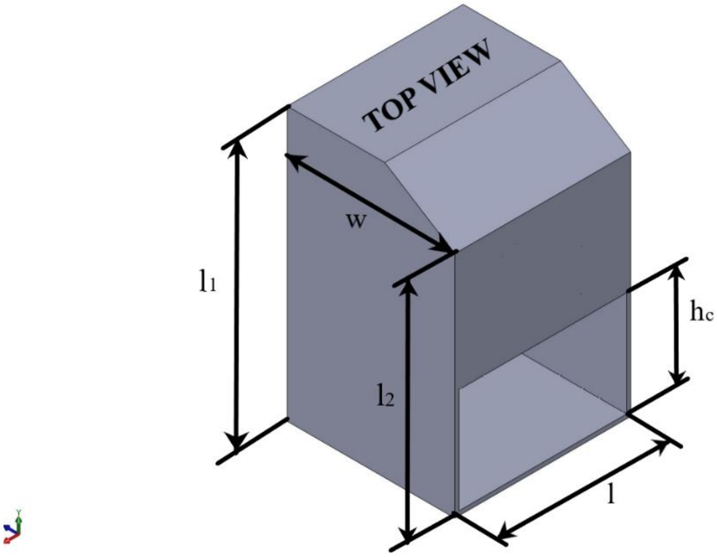

Figure S1. 3D isometric view with parameters

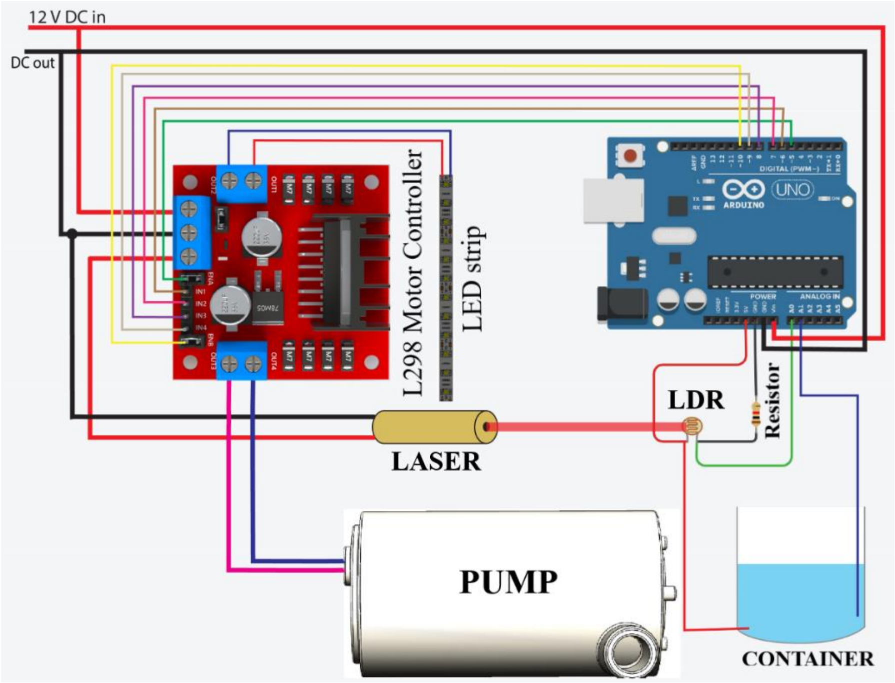

Figure S2 : Schematic of the dispenser circuit

```
#define limit 550 // LDR sensor threshold
void setup()
{
   Serial.begin(115200);
// All motor control pins are outputs
  pinMode(10, OUTPUT);
  pinMode(9, OUTPUT);
  pinMode(8, OUTPUT);
  pinMode(5, OUTPUT);
  pinMode(6, OUTPUT);
  pinMode(7, OUTPUT);
}
void goStraight()
{
  int analogValue = analogRead(A0);
  if (analogValue > limit) //ldr senses the hand
  {
//turn on LED
  digitalWrite(7, LOW);
  digitalWrite(6, HIGH);
// set speed to 150 out 255
  analogWrite(5,255);
// turn on motor A
  digitalWrite(9, HIGH);
  digitalWrite(8, LOW);
// set speed to 150 out 255
  analogWrite(10,10);
//speeding the pump
  for(int i=10;i<255;i=i+2)
  {
    analogWrite(10,i);
    delay(10);
  }
  delay(500);
// now turn off motors
  digitalWrite(9, HIGH);
  digitalWrite(8, LOW);
  analogWrite(10,30);
// now turn off light
```


```
// now turn off motors
  digitalWrite(9, HIGH);
  digitalWrite(8, LOW);
  analogWrite(10,30);
// now turn off light
```


```
  digitalWrite(6, LOW);
  digitalWrite(7, LOW);
  }
  else
  {
  digitalWrite(9, LOW);
  digitalWrite(8, LOW);
  }
  }

void loop()
{
  int analogValue = analogRead(A0);
  Serial.println(analogValue);
  // low-level indication
  if ( analogRead(A1) > 100)
  {
    goStraight();
    delay(2000);
  }
  else
//LED light blinking to indicate
  //sanitizer level lowinside the
    //container
  {
    digitalWrite(7, LOW);
    digitalWrite(6, HIGH);
    delay(1000);
    digitalWrite(7, LOW);
    digitalWrite(6, LOW);
    delay(1000);
  }
}                //End of the code
```

Figure S3 : The coding of the dispenser according to the algorithm
